# Development of machine learning models to predict cancer-related fatigue in Dutch breast cancer survivors up to 15 years after diagnosis

**DOI:** 10.1007/s11764-023-01491-1

**Published:** 2023-12-07

**Authors:** Lian Beenhakker, Kim A. E. Wijlens, Annemieke Witteveen, Marianne Heins, Joke C. Korevaar, Kelly M. de Ligt, Christina Bode, Miriam M. R. Vollenbroek-Hutten, Sabine Siesling

**Affiliations:** 1 Department of Biomedical Signals and Systems, Technical Medical Centre, University of Twente, Postbox 217, 7500 AE Enschede, The Netherlands; 2 Department of Primary Care, Netherlands Institute for Health Services Research (Nivel), Utrecht, The Netherlands; 3 Division of Psychosocial Research and Epidemiology, Netherlands Cancer Institute, Amsterdam, The Netherlands; 4 Department of Psychology, Health and Technology, University of Twente, Enschede, The Netherlands; 5 Board of Directors, Medisch Spectrum Twente, Enschede, The Netherlands; 6 Department of Health Technology and Services Research, Technical Medical Centre, University of Twente, Enschede, The Netherlands; 7 Department of Research and Development, Netherlands Comprehensive Cancer Organization (IKNL), Utrecht, The Netherlands

**Keywords:** Cancer-related fatigue, Breast cancer, Risk prediction, Machine learning, Cancer survivorship

## Abstract

**Purpose:**

To prevent (chronic) cancer-related fatigue (CRF) after breast cancer, it is important to identify survivors at risk on time. In literature, factors related to CRF are identified, but not often linked to individual risks. Therefore, our aim was to predict individual risks for developing CRF.

**Methods:**

Two pre-existing datasets were used. The Nivel-Primary Care Database and the Netherlands Cancer Registry (NCR) formed the Primary Secondary Cancer Care Registry (PSCCR). NCR data with Patient Reported Outcomes Following Initial treatment and Long-term Evaluation of Survivorship (PROFILES) data resulted in the PSCCR-PROFILES dataset. Predictors were patient, tumor and treatment characteristics, and pre-diagnosis health. Fatigue was GP-reported (PSCCR) or patient-reported (PSCCR-PROFILES). Machine learning models were developed, and performances compared using the C-statistic.

**Results:**

In PSCCR, 2224/12813 (17%) experienced fatigue up to 7.6 ± 4.4 years after diagnosis. In PSCCR-PROFILES, 254 (65%) of 390 patients reported fatigue 3.4 ± 1.4 years after diagnosis. For both, models predicted fatigue poorly with best C-statistics of 0.561 ± 0.006 (PSCCR) and 0.669 ± 0.040 (PSCCR-PROFILES).

**Conclusion:**

Fatigue (GP-reported or patient-reported) could not be predicted accurately using available data of the PSCCR and PSCCR-PROFILES datasets.

**Implications for Cancer Survivors:**

CRF is a common but underreported problem after breast cancer. We aimed to develop a model that could identify individuals with a high risk of developing CRF, ideally to help them prevent (chronic) CRF. As our models had poor predictive abilities, they cannot be used for this purpose yet. Adding patient-reported data as predictor could lead to improved results. Until then, awareness for CRF stays crucial.

**Supplementary Information:**

The online version contains supplementary material available at 10.1007/s11764-023-01491-1.

## Introduction

One of the most frequently patient-reported problems after breast cancer diagnosis and treatment is cancer-related fatigue (CRF) [1–3]. If CRF does not reduce in the first 6 months after primary treatment, it is labeled *chronic* CRF [4, 5]. Not all patients experience CRF, and for most, the level of fatigue decreases over time. Still, almost 30% of the patients experience increasing or high levels of fatigue up to 5 years after diagnosis [6]. Fatigue affects physical, cognitive, and emotional functioning of patients [7].

Various non-pharmacological interventions have been found useful in the prevention and reduction of CRF [8–11]. Accordingly, timely identification of patients at high risk of developing (chronic) CRF is important. This allows them to start an intervention to prevent or reduce CRF and prevent it from becoming chronic [4]. So, high-risk patients are either those likely to develop CRF despite not experiencing fatigue yet or those with ongoing fatigue that might not reduce over time.

In literature, factors shown to be associated with CRF included depression [2, 6], anxiety [12–14], baseline fatigue (before treatment) [12, 15], sleeping problems [6, 14], physical inactivity [13], and type of primary treatment (chemotherapy with or without other treatment modalities) [2, 13]. Furthermore, age [13, 14], BMI [6, 14, 15], difficulties with coping with cancer and catastrophizing [16, 17] are recognized as factors related to CRF. Yet, in most of these studies, factors were determined on group-level, and not linked back to individual risks [2, 6, 13, 15, 16]. Two studies used linear models to determine individual CRF risks [12, 14], without taking into account possible unknown interactions between variables.

Instead of linear traditional statistical models, machine learning can be an alternative. Statistical methods are generally known for inference and explaining relationships between variables, while machine learning has the potential to be better for prediction without always providing a precise explanation of the relation between input and output [18, 19]. Machine learning models are also supposed to recognize complex, possibly non-linear, relationships between the variables, potentially leading to better performances [19–21]. This methodology therefore seems a promising alternative, especially given the complexity of CRF.

Machine learning approaches have already been used in multiple oncological settings [22] to predict cancer-related symptoms or care needs [23–26]. Fatigue has been predicted as possible outcome measure by Lee et al. [23] with poor discrimination (*AUC*: 0.60) and by Lindsay et al. [24] with acceptable discrimination (*AUC*: 0.797). This latter study was in a limited patient group after radiotherapy with a mean follow-up period of 2.6 years [24].

In summary, CRF is a problem for many breast cancer survivors. To support those at risk of CRF with an intervention, first, high-risk patients should be identified. While factors associated with CRF have been recognized, they are not often used to determine individual risk. Therefore, this study aims to predict the risk an individual breast cancer patient has for developing CRF. To recognize the possible complexity of CRF, we use machine learning for prediction.

## Methods

### Datasets

The data concerns both primary and secondary care as well as patient-reported data. The Netherlands Institute for Health Service Research (Nivel) collects data of a representative sample of Dutch General Practitioners (GPs) into the Nivel-Primary Care Database (Nivel-PCD). In this database, around 500 GPs are included, covering about 10% of the Dutch population [27]. The Netherlands Comprehensive Cancer Organization (IKNL) collects data directly from the patient files within all hospitals (secondary care) within the Netherlands on all cancer diagnoses and hosts this information as the Netherlands Cancer Registry (NCR) [28]. Lastly, patient-reported data has been collected using the Patient Reported Outcomes Following Initial Treatment and Long-term Evaluation of Survivorship (PROFILES) registry (https://www.profilesregistry.nl/ [29]).

In two previous studies, these registries were used to create two different datasets [1, 30, 31]. For the goal of this study, we could re-use these both datasets. For the first dataset, the NCR and Nivel-PCD were combined to form the Primary Secondary Cancer Care Registry (PSCCR) [30]. For the second dataset, the PROFILES registry was used to distribute questionnaires to a subset of patients in the NCR, combining these two registries into the PSCCR-PROFILES [1, 31]. The combination of the various sources of data into the PSCCR and PSCCR-PROFILES is graphically presented in Online Resource 1. In the next subsections, further details regarding both datasets are described.

#### PSCCR dataset

Patients in the PSCCR were diagnosed with breast cancer between 2000 and 2016 and information on symptoms and diagnoses registered by their GP was available for (a part of) the period of 2008 to 2017. Patients were included if they had GP data available for at least 3 months before their breast cancer diagnosis [30] because of administrative reasons in the Nivel-PCD, where patients are included every quarter of a year.

The outcome measure of fatigue was binary; all patients for whom their GP-registered fatigue symptoms at any point after their breast cancer diagnosis were listed as fatigued; all others formed the non-fatigued group.

Input data for the models were patient, tumor, and treatment characteristics, and pre-diagnosis health. Pre-diagnosis health described the health status of patients *before* breast cancer diagnosis and followed from GP data, including the number of visits to the GP before diagnosis. For each symptom/diagnosis, the GP uses a specific ICPC code (International Classification of Primary Care). As there were 592 different codes, a selection had to be made. Therefore, we checked what percentage of patients experienced each complaint in the total population, the fatigued group, and the non-fatigued patient group. Performing this check on all three groups ascertained us to also select those complaints that occurred more often in one group compared to the other group. Based on the occurrences of complaints, we decided on a threshold to select those symptoms/diagnoses that were experienced by > 3% in at least either of the groups. With this threshold, we selected 32 (5%) of the complaints. Lowering the threshold to 2% would double the complaints included. The ICPC codes related to breast cancer and having no illness were removed. For those ICPC codes that were not selected based on this threshold, but the symptom was reported as factor related to CRF in literature, additional univariable χ^2^ analyses (*α* = 0.05) were performed. With this analysis, we were still able to check how these variables related to fatigue after breast cancer in our dataset.

#### PSCCR-PROFILES dataset

The PSCCR-PROFILES data was collected between September 2017 and March 2018; details are reported elsewhere [1, 31]. In these previous studies, KL collected patient-reported data of 404 patients [1]. The patient-reported data followed from a questionnaire consisting of three parts: (1) The EORTC-QLQ-C30 [32] to measure Health Related Quality of Life, (2) the validated Symptoms and Perceptions (SAP) [33] questionnaire which was extended with breast cancer-specific symptoms, and (3) demographics and disease status.

The outcome measure of fatigue followed from the SAP questionnaire. The main question asked was twofold: “*Which of the following health problems have you experienced over the recent year? And for which of these health problems did you visit a primary care physician or other doctor?*” Fatigue was one of the listed health problems and for both questions, patients could report a binary yes/no answer. Both questions and the reported outcomes by patients were considered relevant for this study. First, based on the answer to the first question, patients were divided into a fatigued and non-fatigued group. Second, the fatigued group was split in fatigued although not visiting a healthcare professional (HCP) and fatigued and visiting professional based on answers to the second question.

Input data for the models included patient, tumor, and treatment characteristics, and baseline characteristics of patients. These baseline characteristics followed from the third part of the questionnaire as described above, with the assumption that these parameters stayed relatively stable over time, e.g., living with partner and/or children or educational level. Answers from the first and second parts of the questionnaire were considered not relevant here, as they described the situation at the time of completing the questionnaire and are not representable for the circumstances at breast cancer diagnosis.

### Prediction models

As fatigue is a complex concept with possible non-linear relationships between predictor variables, machine learning was used for the prediction of fatigue [18, 19]. Various machine learning models were selected based on the different types of models. Models described in previous studies are neural networks or multi-layer perceptron (MLP), decision trees, which can also be extended into a random forest classifier (RFC), support vector machines, which are computationally expensive, Bayesian networks or (Gaussian) Naïve Bayes (GNB), a machine learning version of logistic regression (LR_ML) and K-nearest neighbors (KNN) [22, 34]. The overviews by Kourou et al. [22] and Makaba and Dogo [34] also explain these different techniques. Of these models, MLP, RFC, GNB, LR_ML, and KNN were selected for this study, on the one hand to compare many models, while on the other hand keeping the comparison computationally doable.

### Data handling

To preprocess the data, LB, KW, and AW discussed all variables and their categories. Variables with little to no variation in the categories were excluded, especially if information was also available in other variables, e.g., a binary variable on whether patients had metastases was removed, as we also included tumor stage in which this is included. Also, for some variables, small adjustments were made to the categories to have fewer categories with low occurrence. An example is that staging categories were reduced by removing subcategories per stage. No further predictor selection was performed, the number of observations/patients included in the dataset was larger than the number of predictors in both the PSCCR and the PSCCR-PROFILES (rule of thumb: at least ten observations per predictor).

Some predictors had missing data and were imputed. To prevent high computation times and have valid imputations, predictors were excluded if more than 50% of the data was missing [35]. The remaining predictors with missing data were imputed using Multiple Imputation by Chained Equations (MICE) with Random Forest Imputation [35, 36], resulting in five imputed datasets. The imputation model uses a Random Forest in which missing variables are imputed by using all other variables. To check if the imputation was successful, LB and AW visually compared the distribution over the categories before and after imputation. Details about the implementation in Python are described in Online Resource 2.

Each of the machine learning models has specific settings that have to be tuned; these are the hyperparameter settings. As an example, one of the hyperparameters for the RFC model is the number of decision trees in the random forest. To tune the hyperparameters and find the optimal hyperparameters, and to determine the overall performance of the models, a nested five-fold cross validation was used on each of the imputed datasets [37]. Additionally, this nested five-fold cross validation helped to prevent overfitting and in determining the final model performance. For this latter aspect, unseen test data was needed that is different from the data used to train the models. So, first, data was randomly divided into five equal folds, of which one is set aside as unseen test data (train/test split). Second, the train data was again randomly subdivided into five equal folds. Using a grid search, hyperparameters were validated by using four folds as train data and the fifth as validation (train/validation split) [38]. Using the optimal hyperparameter settings, all train data of the train/test split was used to develop a final model which was tested with the unseen data.

To be able to pool the results of the imputed datasets and the folds of the cross validation, the splits in the five-fold cross validation were the same for each imputed dataset. So, the predictions on the test set for each of the imputations were averaged to get to a pooled prediction per fold of the cross validation [25]. A graphical representation of both the nested fivefold cross validation and the pooling of the imputed data is shown in Online Resource 1.

### Performance measures

Performance of the various models was assessed using the C-statistic or the area under the receiver operator characteristic curve (*AUC*). The *AUC* takes both the true positive rate (TPR) and the false positive rate (FPR) into account. The *AUC* varies between 0 and 1 and based on its specific value, discrimination is poor (0.5–0.7), acceptable (0.7–0.8), excellent (0.8–0.9), and outstanding (0.9–1) [39]. For an *AUC* value equal to or lower than 0.5, there is no discrimination [39]. For reporting the *AUC* values, predictions were not pooled, instead the *AUC* was averaged over twenty-five predictions: five imputed datasets and five folds per dataset. The mean and standard deviation over these twenty-five predictions were reported. The *AUC* value was reported on both the test data as well as on the train data to show the apparent predictive performance of the model to check for overfitting.

Besides the *AUC* value, the predicted probability of each of the models was compared to the true binary values. Additionally, classification plots were used to show how both the TPR and FPR change with varying thresholds [40]. Ideally, from these plots, a threshold can be determined such that the TPR is still high (close to 1) while the FPR is already lower (close to 0). Next to classification plots, calibration plots were developed to check how well the models were calibrated.

A final analysis followed from the RFC model, as this model has the ability to return feature importance leading to an additional analysis. This information was used to assess the importance of each of the variables in the model. For each variable, the importance was averaged over all trees in the RFC and the imputed datasets, and the ten most important features were reported. In case the apparent predictive performance showed large differences between the performance on the train and test set (thus overfitting in the models), fewer variables were selected based on this analysis of the most important features on the RFC to compare the performance using fewer variables.

Above analyses were performed for the PSCCR data, the PSCCR-PROFILES data with two groups (non-fatigued/fatigued) and the PSCCR-PROFILES data with three groups (non-fatigued/fatigued + not visiting HCP/fatigued + visiting HCP). This latter analysis was done using a multiclass OneVsRest classification model.

To report on the development of the prediction models, the Transparent Reporting of a multivariable prediction model for Individual Prognosis or Diagnosis (TRIPOD) checklist [41] was used, as the checklist for artificial intelligence modeling (TRIPOD-AI) is still under development [42]. Online Resource 3 contains the filled-in checklist and information related to checklist items not reported in-text. All analyses were performed in Python, see Online Resource 2 for the version numbers of the used packages.

## Results

### Study population

From the PSCCR dataset, 12,813 breast cancer patients with a registered GP consultation were included, of which 2224 (17%) visited their GP with fatigue complaints after cancer diagnosis. At diagnosis, patients were on average 59 (standard deviation (*SD*): 13) years old. On average, there was follow-up data available for a period of 4.6 (*SD*: 2.3) years after diagnosis. It varied for what period after diagnosis this data was available; on average, there were 7.6 (*SD*: 4.4) years between diagnosis and the end of the follow-up period. Almost all patients received surgery (95%); furthermore, patients received chemotherapy (43%), radiotherapy (67%), and/or hormone therapy (53%). A total of 53 variables were included as predictor from the PSCCR data: 23 described patient, tumor, and treatment characteristics, 30 described pre-diagnosis health and GP visits (see Table 1 or an extended version with all predictors in Online Resource 1).

**Table 1:** Demographics of participants in both datasets

		PSCCR (*n* = 12,813)		PSCCR-PROFILES (*n* = 390)	
Fatigued					
Fatigue complaints at GP		2224 (17.4%)			
SAP-question fatigue				254 (65.1%)	
SAP-question visit professional with fatigue				70 (17.9%)	
Age at diagnosis (mean ± standard deviation)		59 ± 13		58 ± 11	
Topography					
Nipple		67 (0.5%)		2 (0.5%)	
Central portion of breast		809 (6.3%)		18 (4.6%)	
Upper-inner quadrant		1521 (11.9%)		55 (14.1%)	
Lower-inner quadrant		866 (6.8%)		31 (7.9%)	
Upper-outer quadrant		4826 (37.7%)		131 (33.6%)	
Lower-outer quadrant		1044 (8.1%)		28 (7.2%)	
Axillary tail of breast		80 (0.6%)		1 (0.3%)	
Overlapping		3326 (26%)		114 (29.2%)	
Not specified		274 (2.1%)		10 (2.6%)	
Missing		0 (0%)		0 (0%)	
Degree of differentiation					
Low grade		2588 (20.2%)		93 (23.8%)	
Intermediary		5129 (40%)		169 (43.3%)	
High grade		3295 (25.7%)		93 (23.8%)	
Missing		1801 (14.1%)		35 (9%)	
pT (pathologically confirmed T status describing tumor size) – TNM staging					
T0		197 (1.5%)		24 (6.2%)	
T1		7703 (60.1%)		231 (59.2%)	
T2		3596 (28.1%)		109 (27.9%)	
T3		353 (2.8%)		9 (2.3%)	
T4		86 (0.7%)		6 (1.5%)	
In situ		95 (0.7%)			
Missing		783 (6.1%)		11 (2.8%)	
pN (pathologically confirmed N status describing lymphe nodes) – TNM staging					
N0		7163 (55.9%)		254 (65.1%)	
N1		3434 (26.8%)		101 (25.9%)	
N2		638 (5%)		19 (4.9%)	
N3		343 (2.7%)		6 (1.5%)	
Missing		1235 (9.6%)		10 (2.6%)	
Tumor stage — TNM staging					
Stage 0		102 (0.8%)			
Stage 1		5812 (45.4%)		179 (45.9%)	
Stage 2		5124 (40%)		166 (42.6%)	
Stage 3		1376 (10.7%)		45 (11.5%)	
Stage 4		353 (2.8%)			
Missing		46 (0.4%)		0 (0%)	
Positive lymph nodes					
None		7545 (58.9%)		253 (64.9%)	
1–3		3574 (27.9%)		113 (29%)	
More than 3		1093 (8.5%)		23 (5.9%)	
Missing		601 (4.7%)		1 (0.3%)	
Chemotherapy					
No		7364 (57.5%)		192 (49.2%)	
Pre-surgery				71 (18.2%)	
Post-surgery				126 (32.3%)	
Pre + post-surgery				1 (0.3%)	
Undefined pre/post		5449 (42.5%)			
Missing		0 (0%)		0 (0%)	
Hormonal therapy					
No		5984 (46.7%)		161 (41.3%)	
Post-surgery				226 (57.9%)	
Pre + post-surgery				3 (0.8%)	
Undefined pre/post		6829 (53.3%)			
Missing		0 (0%)		0 (0%)	
Targeted therapy					
No		11,832 (92.3%)		342 (87.7%)	
Pre-surgery				1 (0.3%)	
Post-surgery				28 (7.2%)	
Pre + post-surgery				19 (4.9%)	
Undefined pre/post		981 (7.7%)			
Missing		0 (0%)		0 (0%)	
Radiotherapy					
No		4240 (33.1%)		102 (26.2%)	
Post-surgery				288 (73.8%)	
Undefined pre/post		8573 (66.9%)			
Missing		0 (0%)		0 (0%)	
Educational level					
Primary education				22 (5.6%)	
Secondary education				90 (23.1%)	
Secondary vocational education				169 (43.3%)	
Higher education				106 (27.2%)	
Missing				3 (0.8%)	
School/work situation					
Going to school/studying				2 (0.5%)	
Paid work				154 (39.5%)	
Unemployed/looking for work				15 (3.8%)	
Incapacitated				18 (4.6%)	
Housewife				45 (11.5%)	
Retired				144 (36.9%)	
Missing				12 (3.1%)	
Still receiving treatment?					
No				176 (45.1%)	
Yes, hormonal therapy				168 (43.1%)	
Yes, other therapy				29 (7.4%)	
Missing				17 (4.4%)	
Radicality of excision at first surgery					
Invasive tumor	DCIS				
Radical/not present	Radical/not present	6406 (50%)			
Radical/not present	Focal/not radical	215 (1.7%)			
Radical/not present	Not radical	127 (1%)			
Focal not radical	Radical/not present	350 (2.7%)			
Focal not radical	Focal not radical	43 (0.3%)			
Focal not radical	Not radical	20 (0.2%)			
Not radical	N/A	328 (2.6%)			
Missing		532 (41.6%)			
Radicality of excision at last surgery					
Invasive tumor	DCIS				
Radical/not present	Radical/not present	6890 (53.8%)			
Radical/not present	Focal/not radical	183 (1.4%)			
Radical/not present	Not radical	48 (0.4%)			
Focal not radical	Radical/not present	261 (2%)			
Focal not radical	Focal not radical	21 (0.2%)			
Focal not radical	Not radical	5 (0%)			
Not radical	N/A	101 (0.8%)			
Missing		5304 (41.4%)			
Social-economic status					
Low		3833 (29.9%)			
Middle		4945 (38.6%)			
High		3968 (31%)			
Missing		67 (0.5%)			
Sentinel node procedure					
Not performed		3125 (24.4%)			
Performed		7856 (61.3%)			
Missing		1832 (14.3%)			
Result of sentinel node procedure					
Negative		5141 (40.1%)			
ITC (≤ 0.2 mm)		455 (3.6%)			
Micro metastases (> 0.2 mm, ≤ 2 mm)		683 (5.3%)			
Positive (> 2 mm)		1505 (11.7%)			
Not found		215 (1.7%)			
Missing		4814 (37.6%)			
Visits to GP (mean ± standard deviation)		16 ± 36			
Complaints before diagnosis, 5 most common		1. Uncomplicated hypertension (*n* = 1233, 9.6%) 2. Cystitis/other urinary infection (*n* = 914, 7.1%) 3. Cough (*n* = 794, 6.2%) 4. Upper respiratory infection acute (*n* = 626, 4.9%) 5. Excessive ear wax (*n* = 606, 4.7%)			

Of the 404 patients in the PSCCR-PROFILES dataset that completed the questionnaire, 390 filled out the SAP-fatigue question. Of these patients, 254 (65%) were fatigued and 70 (18%) reported to have visited a healthcare professional for their fatigue complaints. By inclusion in the PSCCR-PROFILES dataset, all patients had surgery. Just more than half (51%) of the patients received (neo)-adjuvant chemotherapy and 74% received radiotherapy. Patients reported that they mostly lived together with their partner (84%), that they either did paid work (40%) or were retired (37%), and that if they had children, their children were living away from home (58%). In the PSCCR-PROFILES, patients completed the questionnaire on average 3.4 (*SD*: 1.4) years after diagnosis. A total of 23 variables were included as predictor from the PSCCR-PROFILES data: eighteen were related to patient, tumor, and treatment characteristics, and five followed from self-reported demographics (see Table 1 or an extended version with all predictors in Online Resource 1).

The percentage of missing data for each variable is reported in Table 1. The missing data patterns for both the PSCCR and the PSSCR-PROFILES dataset are reported in Online Resource 1. Visual comparison of the distribution over the categories of the non-imputed and imputed variables showed these datasets were comparable. In general, variables with more missing values had fewer matching distributions between the datasets. For PSCCR, these were menopausal status, radicality of excision at first and last surgery, pT status (pathologically confirmed T status describing tumor size) of TNM staging and result of sentinel node procedure; for PSCCR-PROFILES, this was the case for menopausal status and pT status of TNM staging.

### Prediction machine learning models

Fatigue was poorly predicted by all prediction models. The *AUC* values (*mean* ± *SD*) varied from 0.504 ± 0.017 to 0.561 ± 0.006 in the PSCCR model and from 0.578 ± 0.083 to 0.669 ± 0.040 in the PSCCR-PROFILES model (two groups, non-fatigued/fatigued, Table 2). Additionally, the multiclass OneVsRest classification with the three groups (non-fatigued/fatigued + not visiting HCP/fatigued + visiting HCP) in the PSCCR-PROFILES data did not show improved results with *AUC* values of 0.505 ± 0.035 to 0.602 ± 0.039 (Table 2). The LR_ML model was the best in all cases. As the multiclass OneVsRest model in the PSCCR-PROFILES dataset did not give improved results compared to the binary classification; further results are only reported for the binary classification.

**Table 2:** Model performance measured with area under the curve (*AUC*) values for the various models and the various datasets. The PSCCR-PROFILES is used in two settings, a binary classification of fatigue and an OneVsRest classification with fatigue and reporting fatigue at a healthcare professional. The PSCCR only has information on GP visits which is used for binary classification. The values are the means and standard deviations over the five folds

		*AUC* values		
		PSCCR-PROFILES	PSCCR-PROFILES	PSCCR
		Binary classification	OneVsRest	
Random forest classifier	Test Train	0.642 ± 0.040 0.826 ± 0.019	0.570 ± 0.038 0.847 ± 0.012	0.556 ± 0.011 0.893 ± 0.016
Logistic regression	Test Train	0.669 ± 0.040 0.712 ± 0.007	0.576 ± 0.032 0.682 ± 0.017	0.561 ± 0.006 0.589 ± 0.003
Gaussian Naïve Bayes	Test Train	0.665 ± 0.036 0.706 ± 0.008	0.602 ± 0.039 0.691 ± 0.012	0.544 ± 0.012 0.553 ± 0.004
K-nearest neighbors	Test Train	0.580 ± 0.044 0.874 ± 0.121	0.505 ± 0.035 0.793 ± 0.104	0.504 ± 0.017 0.800 ± 0.083
Multi-layer perceptron	Test Train	0.578 ± 0.083 0.737 ± 0.005	0.555 ± 0.043 0.596 ± 0.009	0.531 ± 0.027 0.549 ± 0.034

The apparent predictive performance of the models on the train data shows that the RFC and the KNN model are overfitting (Table 2). However, selecting fewer variables as predictor did not improve the performance of the models on the test data to acceptable *AUC* values (*AUC* > 0.7). These performances are reported in Online Resource 1 for reference.

**Fig. 1:**
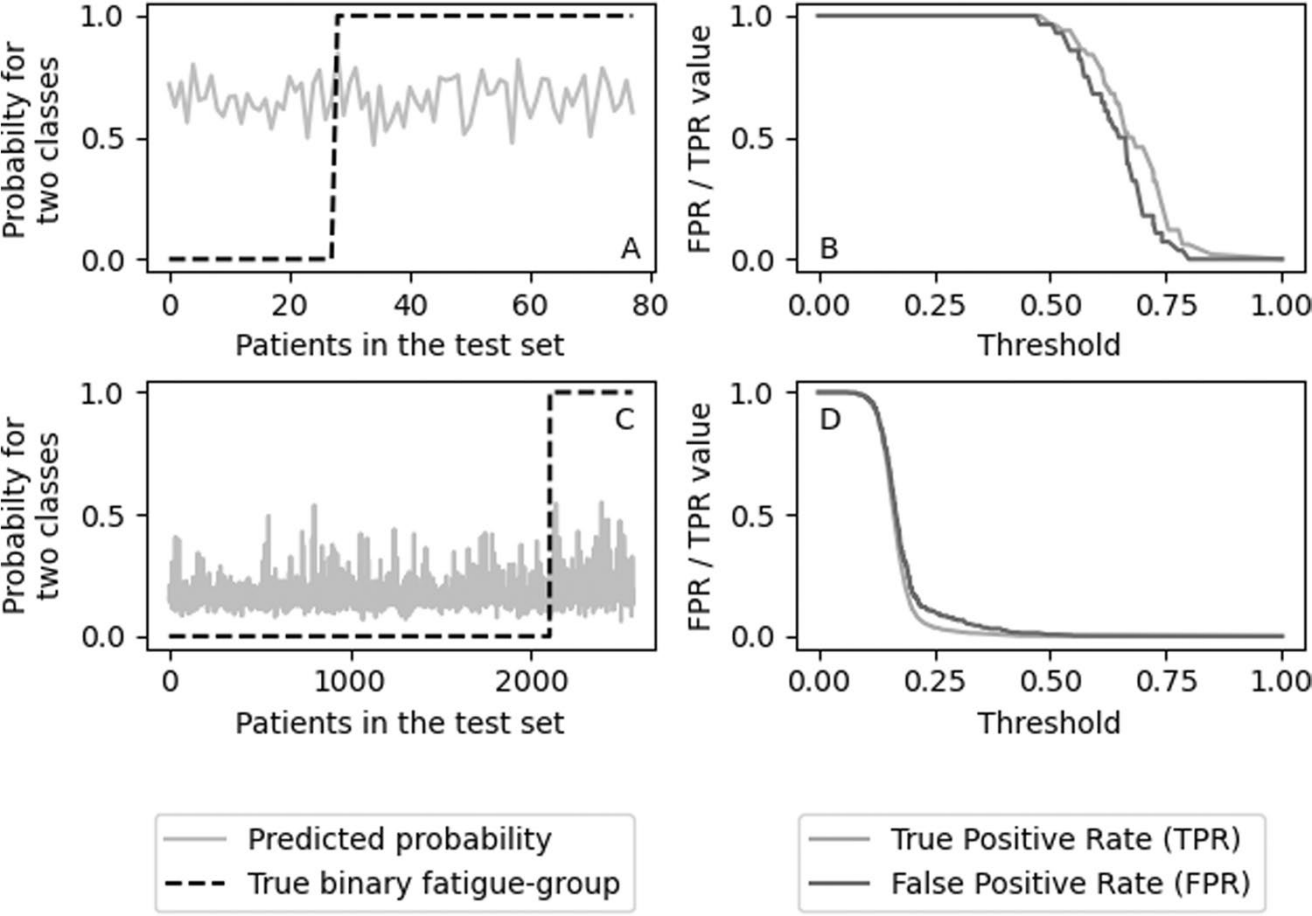
Results of the pooled predictions of the first fold with the best model per dataset (LR_ML for both datasets). **A** PSCCR-PROFILES data, the gray line shows the predicted risk for each individual in the test set, whereas the dashed black line shows the true value (non-fatigued [0] or fatigued [1]. **B** Classification plot of PSCCR-PROFILES data, the false positive rate (FPR), and true positive rate (TPR) for varying thresholds. **C** PSCCR data, the gray line shows the predicted risk for each individual in the test set, whereas the dashed black line shows the true value (non-fatigued [0] or fatigued [1]. **D** Classification plot of PSCCR data, the false positive rate (FPR), and true positive rate (TPR) for varying thresholds.

When comparing the results of the prediction against the true values, these plots show that the predicted probability for fatigue is similar for the fatigued and non-fatigued groups (Fig. 1, left panels). The classification plots show that no threshold can be set such that the FPR is low and TPR is still high (Fig. 1, right panels). The calibration plots showed that the models are also not well calibrated. These plots are reported for the various models in Online Resource 1.

The three most important features in the PSCCR data were total number of visits to the GP before diagnosis of breast cancer, topography/location of the tumor in the breast, and age at diagnosis (Table 3). Also, related to the complaints patients had before breast cancer diagnosis, fatigue was among the ten most important features, and thus was the most relevant complaint before diagnosis to predicting fatigue (Table 3). For the PSCCR-PROFILES data, the three most important features were chemotherapy, school/work situation, and still receiving treatment (Table 3).

**Table 3:** Results of the important feature analysis for the RFC model. The ten most important features are listed in the table below

Important features PSCCR-PROFILES	Important features PSCCR
1. Chemotherapy 2. School/work situation 3. Still receiving therapy (e.g., hormonal) 4. Topography/location in breast 5. Positive lymph nodes 6. Age at diagnosis 7. Tumor stage 8. Educational level 9. pN (TNM staging) 10. Degree of differentiation	1. Visits to GP 2. Topography/location in breast 3. Age at diagnosis 4. Social economic status 5. Radicality of excision at first surgery 6. Result of sentinel node procedure 7. Degree of differentiation 8. Radicality of excision at last surgery 9. Complaints before diagnosis — fatigue 10. pT (TNM staging)

The additional univariable χ^2^ analyses were performed for depression and anxiety [2, 6, 12–14]. Both complaints did not have a single ICPC code in the PSCCR dataset. Depression has two codes (“depressive disorder” and “feeling depressed”), and the univariable χ^2^ analyses showed that both are not significantly related with fatigue. Anxiety has 38 ICPC codes and the univariable χ^2^ analyses showed that only one of those codes was significantly related to fatigue (“feeling anxious/nervous/tense/inadequate”, *p* = 0.010).

## Discussion

In this study, we aimed to predict the risk of developing CRF for an individual breast cancer patient to enable early CRF interventions and prevent CRF of becoming chronic. For this, we used patient, tumor, and treatment characteristics, pre-diagnosis health, and self-reported baseline characteristics. Risk was predicted using machine learning models, as this is a suitable method for predictions [18, 19]. Our results showed that, from the PSCCR and PSCCR-PROFILES datasets, the risk for CRF cannot be predicted accurately, as we found poor discriminative values (*AUC* < 0.7) for all models in both datasets.

There could be several reasons for the poor predictive ability of the models. Machine learning methodology should be able to find complex, non-linear associations between the variables [19–21]. From our study, it is unclear if such associations were present in the data, and the models were unable to find them, or if fatigue is unrelated to patient, tumor, and treatment characteristics, pre-diagnosis health, and self-reported demographics. Below, we will discuss the input data and outcome measure and their possible relation to the poor discriminative ability of our models.

### Input data

The input data followed from several sources and described clinical data (NCR), pre-diagnosis health (Nivel-PCD) and self-reported demographics (PROFILES). In other studies that predicted fatigue with machine learning, predictors also followed from clinical data [24] or clinical data extended with genetic data [23]. Of those, only Lindsay et al. [24] found improved results, with acceptable discrimination (*AUC*: 0.797), but in a limited, homogenous, participant group who all received radiotherapy and had a median follow-up period of 2.6 years. Our population was a representative sample of the Dutch breast cancer population with follow-up data up to 15 years after diagnosis. Even though machine learning should be able to identify complex patterns, it could be that our patient group was too heterogeneous. Dividing the dataset into subsets might have been a solution; however, this would also have decreased the sample size, while machine learning models need a large dataset.

The variables that were most important in the RFC model (Table 3) can be compared to previously reported factors related to CRF. In literature, depression [2, 6], anxiety [12–14], baseline fatigue [12, 15], sleeping problems [6, 14], physical inactivity [13], type of primary treatment (chemotherapy with or without other treatment modalities) [2, 13], age [13, 14], BMI [6, 14, 15], difficulties with coping with cancer and catastrophizing [16, 17] were found to correlate with fatigue. We also found chemotherapy and age as most influential factors, and baseline fatigue had most impact of all pre-diagnosis health symptoms (Table 3). Depression and anxiety relate to pre-diagnosis health; however, both were not included because less than 3% of the patients reported these complaints at their GP. This is comparable to the general Dutch population [43], although most likely more patients experienced depression and anxiety, but did not report this at their GP. It is important to note that these results should be interpreted with caution due to the poor discriminative ability of the models.

To improve the input data, more information regarding the abovementioned factors should be included. Most of them can follow from patient-reported outcomes measures (PROMs), e.g., depression, anxiety, sleeping problems, and current ways of coping. PROMs have already been implemented in clinical settings [44]; however, the use of PROMs in prediction with machine learning is still a relatively new research area [45].

### Output measure

The use of patient-reported data is also relevant to measure fatigue as outcome measure. In the two datasets included in our study, fatigue followed from GP-reported data (PSCCR, 17% fatigued) and patient-reported data (PSCCR-PROFILES, 65% fatigued). Lindsay et al. [24] used clinician-reported data (59% fatigued) automatically extracted from patients’ medical records at a radiotherapy institution. Patients are less likely to report cancer-related problems to their GP [46] and prefer to report to their breast cancer specialist in follow-up care [47]. Furthermore, there is a discrepancy between patient-reported outcomes and clinician-reported outcomes as clinicians tend to underestimate, and with that underreport, complaints of cancer patients [48, 49]. Information might therefore be missing and fatigue underreported in the PSCCR dataset. This is also supported by the PSCCR-PROFILES dataset, as 65% of the patients reported to be fatigued and only 18% reported to also have visited a healthcare professional for these complaints. Using patient-reported data for the outcome measure might therefore result in a better division in the fatigued and non-fatigued group, despite the risk of recall bias of patient-reported data.

The model performances of the PSCCR and PSCCR-PROFILES also hint towards patient-reported data being better than GP-reported data. The best performing model for the PSCCR data had an *AUC* of 0.561, whereas the best performing model for the PSCCR-PROFILES data did better with an *AUC* of 0.669. Of note, there are also other factors that might have caused the difference. First, the models have different input data, both use data of the NCR, in the PSCCR pre-diagnosis health is included, whereas PSCCR-PROFILES has self-reported demographics. Second, PSCCR-PROFILES has a smaller sample size (390 patients), resulting in a higher risk of overfitting.

Another reason for the poor discriminative abilities is that fatigue is a multidimensional and complex complaint which we measured in a binary way. Lee et al. [23] measured and predicted fatigue dimensions (physical, emotional, and cognitive fatigue) using clinical and genetic data but found no improved results compared to our study (best *AUC*: 0.60 for cognitive fatigue [23]). For this study, the fact that we could not measure fatigue dimensions may not have influenced our results much. Still, when expanding the input data with patient-reported data, it would be interesting to see if it is possible to predict different dimensions. This might be relevant to patients, as well as recommendations for an intervention for CRF.

### Strength and limitations

Our study has some strengths and limitations. One of the strengths is the large and comprehensive study population of the PSCCR group, in which over 12,000 patients were included. The NCR collects data from every cancer diagnosis [28], and Nivel data is also collected for a considerable number of representative GPs [27], making the PSCCR data representative for the Dutch population. Both databases have an opt-out procedure for patients, but few patients are removed from the registries, making the risk of selection bias very small. Therefore, our results would have been generalizable to the Dutch breast cancer population.

Another strength is the use of two datasets to predict fatigue, PSCCR and PSCCR-PROFILES. This gave us the opportunity to compare and contrast these two and their results within our study. They differ in the measurement of fatigue, while they have overlap in input data, making internal comparative conclusions more robust than an external comparison.

The use of GP-reported data allowed us to include over 12,000 patients; however, a limitation is that fatigue is probably not measured accurately as not all patients might report their fatigue complaints at their GP. Also, follow-up information of patients is not available over the full follow-up period, both in PSCCR and PSCCR-PROFILES. In the PSCCR, it depended on the period in which patients were enrolled at the specific GP practice, and in the PSCCR-PROFILES, patients were asked to report for the last year cross-sectionally. In both cases, the chronicity of CRF is not reflected in the outcome measure, and we had a heterogeneous outcome measure of fatigue. On the one hand, it might be that we missed patients that should have been included in the fatigued group, and, on the other hand, it might also be that not all reported fatigue was *cancer-related* fatigue.

Another limitation is related to the use of the feature importance of the RFC model. First, as the *AUC* values of the RFC models do not show good discriminative ability, it is important that these results are interpreted with caution. Second, the information was only available for the RFC model and is not one-to-one transferable to the other models. It is questionable if knowledge of important features can be transferred between the models, i.e., in other models, other features might have more impact on the prediction [50, 51]. Lastly, the feature importance does not show the direction of the effect. This is in line with machine learning being better for prediction without being able to explain the relation between in- and output variables [18].

### Future study directions and implications

As mentioned, both input data and outcome measures could benefit from adding data reported by patients themselves, for example related to pre-diagnosis health and current health status. When using this information to predict, it is important to consider at what moment this prediction takes place and what patient-reported information is available at that specific moment in time.

In this study, we did not find models that can predict the risk of fatigue accurately. In future studies where models with a higher discriminative ability are developed, it is also important to think of how to implement these models in healthcare. For this, it is important to determine how risks are reported to patients, that is, do patients receive the risk as a value between 0 and 100% or are they classified as high-risk or low-risk patients. In the latter case, an optimal cut-off point should be identified, for example with the Youden index [52]. Also, the models should be explainable to both the clinician and the patient [53].

For now, it is important to further increase the awareness for CRF, both for healthcare professionals and patients. Patients do not always report their complaints to their GP or another healthcare professional [1] because they think CRF is inevitable and feel not supported [54]. However, if both patients and healthcare professionals are more aware and know there are interventions available, patients might share their struggle more often. Consequently, more patients can then be supported with an intervention for fatigue [14] which can, after future studies, also be personalized based on patient preferences [55].

## Conclusion

The goal of this study was to predict the individual risk for CRF to enable identification of patients with a high risk for CRF. For this purpose, we used various machine learning models. Our results showed that neither using data from primary and secondary care (PSCCR) nor using data from secondary care combined with patient-reported data (PSCCR-PROFILES), was it possible to accurately predict CRF. The use of patient-reported fatigue led to higher *AUC* values than GP-reported fatigue, stressing the importance of PROMs. As these data were only available as output, future research should show if PROMs can be used as predictors to determine individual risk for CRF.

Following our study, it is not yet possible to identify individual patients at risk of developing CRF. Still, it is important to support these patients with an early intervention for CRF to prevent it of becoming chronic. Therefore, it is important that both patients and healthcare professionals become and stay aware of CRF and the complexity of this long-term effect after (breast) cancer.

## Supplementary Information

Below is the link to the electronic supplementary material.

Supplementary file1 (PDF 845 KB)

Supplementary file2 (PDF 337 KB)

## Data Availability

Aggregated data of the NCR is available at https://iknl.nl/nkr-cijfers. It is possible to request data of the NCR on record level via https://iknl.nl/en/ncr/apply-for-data. Data of the PSCCR can be requested at IKNL and Nivel via the PSCCR project-group by contacting Nivel via zorgregistraties@nivel.nl. Data of the PSCCR-PROFILES data can be requested at IKNL via gegevensaanvraag@iknl.nl.
