# Supplementary material for: Development of machine learning models to predict cancer-related fatigue in Dutch breast cancer survivors up to 15 years after diagnosis
Source: J Cancer Surviv. 2023 Dec 7;19(2):580–93. doi: 10.1007/s11764-023-01491-1 (PMC11926043; doi:10.1007/s11764-023-01491-1)
Supplement: Supplementary file 1 — Supplementary file1 (PDF 845 KB) [file 11764_2023_1491_MOESM1_ESM.pdf]

# Development of machine learning models to predict cancer-related fatigue in Dutch breast cancer survivors up to 15 years after diagnosis

Lian Beenhakker, Kim A.E. Wijlens, Annemieke Witteveen, Marianne Heins, Joke C. Korevaar, Kelly M. de Ligt, Christina Bode, Miriam M.R Vollenbroek-Hutten, Sabine Siesling

Correspondence to:

Annemieke Witteveen
Department of Biomedical Signals and Systems,
University of Twente,
Postbox 217, 7500 AE, Enschede,
The Netherlands
a.witteveen@utwente.nl
+31 53 489 4708

## Supplementary material

### Online Resource 1: Additional tables and figures

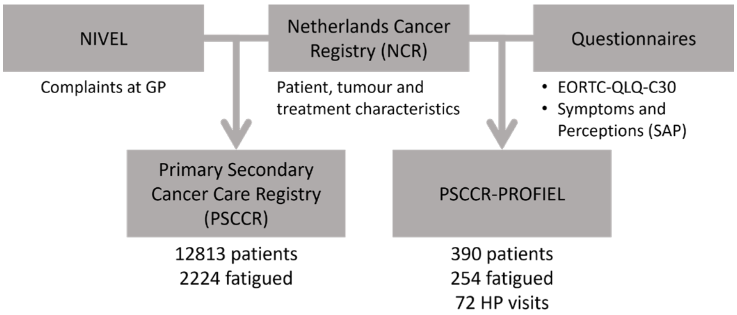


*Figure 1 – Combination of three different databases to create the two datasets used to predict CRF.*

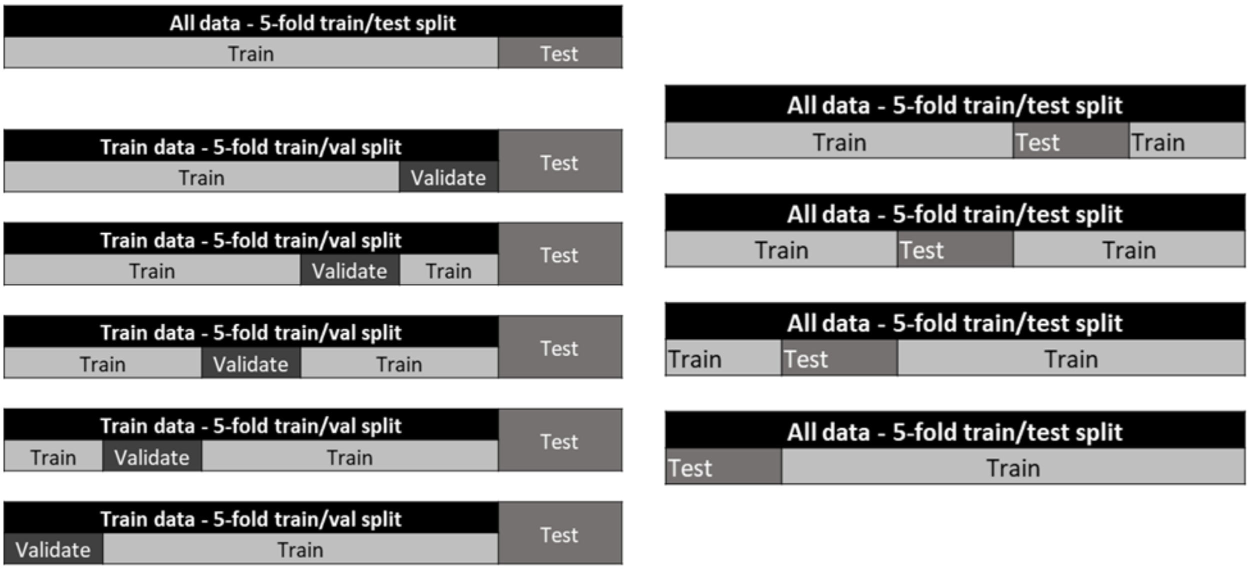


*Figure 2 – Graphical representation of the nested 5-fold cross validation. On the left: Data is first split into a train and test batch, after which the train data is split again into a train and validation batch. Based on the five validations, the optimal hyperparameter settings are determined to develop a model on all train data. This model is then tested using the test batch left out at the beginning. On the right: This process is repeated another four times, as the first split divided the data into five.*

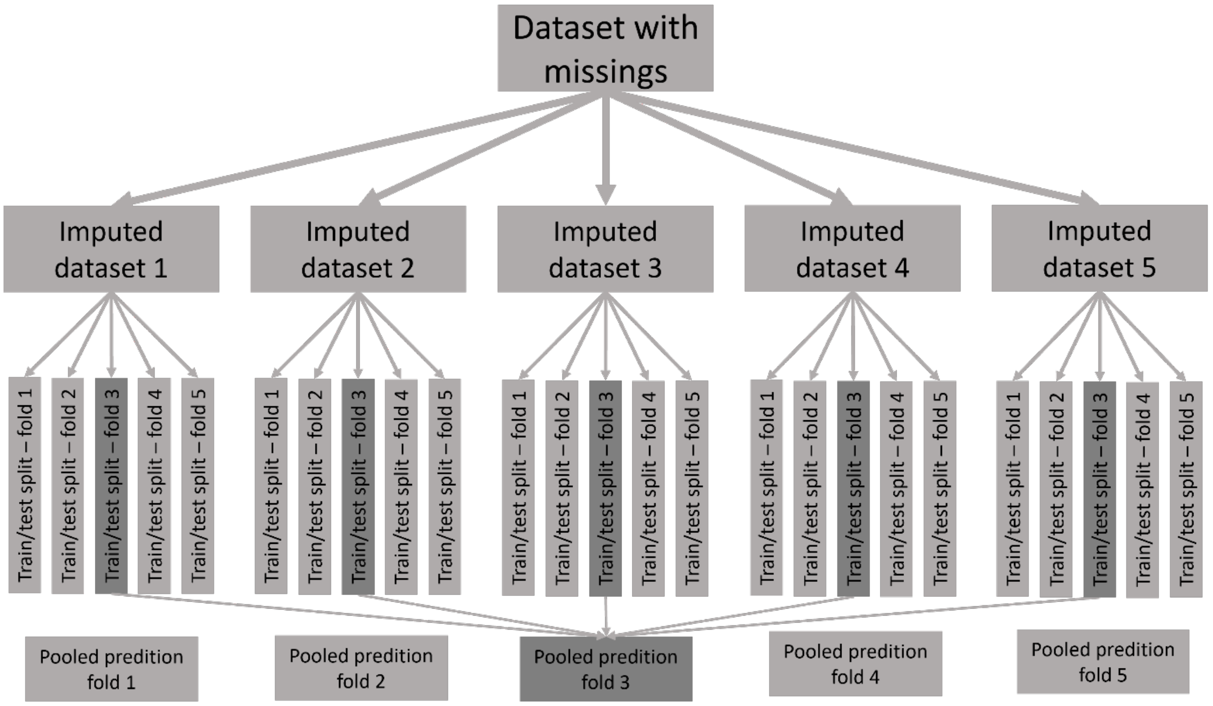


*Figure 3 – Graphical representation of the imputation and pooling. The data with missing values is imputed resulting into five imputed datasets. Each dataset is split into five train/test folds using the same split for every imputed dataset. For the pooling, the overlapping folds are combined to end into a pooled prediction per fold.*

*Table 1 – Extended version of demographics of participants in both datasets.*

| | PSCCR (n=12,813) | | PSCCR-PROFILES (n=390) | |
|---|---|---|---|---|
| Fatigued | | | | |
| Fatigue complaints at GP | 2224 | (17.4%) | | |
| SAP-question fatigue | | | 254 | (65.1%) |
| SAP-question visit professional with fatigue | | | 70 | (17.9%) |
| Age (mean ± standard deviation) | 59 ± 13 | | 58 ± 11 | |
| Menopausal status | | | | |
| Premenopausal | 1180 | (9.2%) | 75 | (19.2%) |
| Perimenopausal | 269 | (2.1%) | 22 | (5.6%) |
| Postmenopausal | 7798 | (60.9%) | 268 | (68.7%) |
| Missing | 3566 | (27.8%) | 25 | (6.4%) |
| Lateralization | | | | |
| Left | 6498 | (50.7%) | 194 | (49.7%) |
| Right | 6299 | (49.2%) | 196 | (50.3%) |
| Missing | 16 | (0.1%) | 0 | (0%) |
| Topography | | | | |
| Nipple | 67 | (0.5%) | 2 | (0.5%) |
| Central portion of breast | 809 | (6.3%) | 18 | (4.6%) |
| Upper-inner quadrant | 1521 | (11.9%) | 55 | (14.1%) |
| Lower-inner quadrant | 866 | (6.8%) | 31 | (7.9%) |
| Upper-outer quadrant | 4826 | (37.7%) | 131 | (33.6%) |
| Lower-outer quadrant | 1044 | (8.1%) | 28 | (7.2%) |
| Axillary tail of breast | 80 | (0.6%) | 1 | (0.3%) |
| Overlapping | 3326 | (26%) | 114 | (29.2%) |
| Not specified | 274 | (2.1%) | 10 | (2.6%) |
| Missing | 0 | (0%) | 0 | (0%) |
| Morphology | | | | |
| Ductal carcinoma | 9560 | (74.6%) | 299 | (76.7%) |
| Lobular carcinoma | 1418 | (11.1%) | 54 | (13.8%) |
| Other | 1835 | (14.3%) | 37 | (9.5%) |
| Missing | 0 | (0%) | 0 | (0%) |
| Degree of differentiation | | | | |
| Low grade | 2588 | (20.2%) | 93 | (23.8%) |
| Intermediary | 5129 | (40%) | 169 | (43.3%) |
| High grade | 3295 | (25.7%) | 93 | (23.8%) |
| Missing | 1801 | (14.1%) | 35 | (9%) |
| Multifocality | | | | |
| No multifocal tumor | 9156 | (71.5%) | 304 | (77.9%) |
| Multifocal tumor | 1949 | (15.2%) | 82 | (21%) |
| Missing | 1708 | (13.3%) | 4 | (1%) |
| pT (pathologically confirmed T status describing tumor size) – TNM staging | | | | |
| T0 | 197 | (1.5%) | 24 | (6.2%) |
| T1 | 7703 | (60.1%) | 231 | (59.2%) |
| T2 | 3596 | (28.1%) | 109 | (27.9%) |
| T3 | 353 | (2.8%) | 9 | (2.3%) |
| T4 | 86 | (0.7%) | 6 | (1.5%) |
| In Situ | 95 | (0.7%) | | |
| Missing | 783 | (6.1%) | 11 | (2.8%) |
| pN (pathologically confirmed N status describing lymphe nodes) – TNM staging | | | | |
| N0 | 7163 | (55.9%) | 254 | (65.1%) |
| N1 | 3434 | (26.8%) | 101 | (25.9%) |
| N2 | 638 | (5%) | 19 | (4.9%) |
| N3 | 343 | (2.7%) | 6 | (1.5%) |
| Missing | 1235 | (9.6%) | 10 | (2.6%) |
| Tumor stage – TNM staging | | | | |
| Stage 0 | 102 | (0.8%) | | |
| Stage 1 | 5812 | (45.4%) | 179 | (45.9%) |
| Stage 2 | 5124 | (40%) | 166 | (42.6%) |
| Stage 3 | 1376 | (10.7%) | 45 | (11.5%) |
| Stage 4 | 353 | (2.8%) | | |
| Missing | 46 | (0.4%) | 0 | (0%) |
| Positive lymph nodes | | | | |
| None | 7545 | (58.9%) | 253 | (64.9%) |
| 1-3 | 3574 | (27.9%) | 113 | (29%) |
| More than 3 | 1093 | (8.5%) | 23 | (5.9%) |

| | | | | |
|---|---|---|---|---|
| Missing | 601 | (4.7%) | 1 | (0.3%) |
| Estrogen | | | | |
| Negative | 1700 | (13.3%) | 64 | (16.4%) |
| Positive | 9363 | (73.1%) | 324 | (83.1%) |
| Missing | 1750 | (13.7%) | 2 | (0.5%) |
| Progesterone | | | | |
| Negative | 3340 | (26.1%) | 113 | (29%) |
| Positive | 7569 | (59.1%) | 274 | (70.3%) |
| Missing | 1904 | (14.9%) | 3 | (0.8%) |
| Her2 | | | | |
| Negative | 8296 | (64.7%) | 322 | (82.6%) |
| Positive | 1404 | (11%) | 58 | (14.9%) |
| Missing | 3113 | (24.3%) | 10 | (2.6%) |
| Chemotherapy | | | | |
| No | 7364 | (57.5%) | 192 | (49.2%) |
| Pre surgery | | | 71 | (18.2%) |
| Post surgery | | | 126 | (32.3%) |
| Pre + post surgery | | | 1 | (0.3%) |
| Undefined pre/post | 5449 | (42.5%) | | |
| Missing | 0 | (0%) | 0 | (0%) |
| Hormonal therapy | | | | |
| No | 5984 | (46.7%) | 161 | (41.3%) |
| Post surgery | | | 226 | (57.9%) |
| Pre + post surgery | | | 3 | (0.8%) |
| Undefined pre/post | 6829 | (53.3%) | | |
| Missing | 0 | (0%) | 0 | (0%) |
| Targeted therapy | | | | |
| No | 11832 | (92.3%) | 342 | (87.7%) |
| Pre surgery | | | 1 | (0.3%) |
| Post surgery | | | 28 | (7.2%) |
| Pre + post surgery | | | 19 | (4.9%) |
| Undefined pre/post | 981 | (7.7%) | | |
| Missing | 0 | (0%) | 0 | (0%) |
| Radiotherapy | | | | |
| No | 4240 | (33.1%) | 102 | (26.2%) |
| Post surgery | | | 288 | (73.8%) |
| Undefined pre/post | 8573 | (66.9%) | | |
| Missing | 0 | (0%) | 0 | (0%) |
| Educational level | | | | |
| Primary education | | | 22 | (5.6%) |
| Secondary education | | | 90 | (23.1%) |
| Secondary vocational education | | | 169 | (43.3%) |
| Higher education | | | | |
| Missing | | | 106 | (27.2%) |
| | | | 3 | (0.8%) |
| Living with partner | | | | |
| Yes | | | 328 | (84.1%) |
| No | | | 59 | (15.1%) |
| Missing | | | 3 | (0.8%) |
| Children | | | | |
| Yes, living at home | | | 103 | (26.4%) |
| Yes, away from home | | | 225 | (57.7%) |
| No children | | | 59 | (15.1%) |
| Missing | | | 3 | (0.8%) |
| School/work situation | | | | |
| Going to school/studying | | | 2 | (0.5%) |
| Paid work | | | | |
| Unemployed / looking for work | | | 154 | (39.5%) |
| Incapacitated | | | 15 | (3.8%) |
| Housewife | | | | |
| Retired | | | 18 | (4.6%) |
| Missing | | | 45 | (11.5%) |
| | | | 144 | (36.9%) |
| | | | 12 | (3.1%) |
| Still receiving treatment? | | | | |
| No | | | 176 | (45.1%) |
| Yes, hormonal therapy | | | 168 | (43.1%) |
| Yes, other therapy | | | 29 | (7.4%) |
| Missing | | | 17 | (4.4%) |
| Radicality of excision at first surgery | | | | |

| | | |
|---|---|---|
| Invasive tumor DCIS | | |
| Radical/not present Radical/not present | 6406 (50%) | |
| Radical/not present Focal/not radical | 215 (1.7%) | |
| Radical/not present Not radical | 127 (1%) | |
| Focal not radical Radical/not present | 350 (2.7%) | |
| Focal not radical Focal not radical | 43 (0.3%) | |
| Focal not radical Not radical | 20 (0.2%) | |
| Not radical N/A | 328 (2.6%) | |
| Missing | 5324 (41.6%) | |
| Radicality of excision at last surgery | | |
| Invasive tumor DCIS | | |
| Radical/not present Radical/not present | 6890 (53.8%) | |
| Radical/not present Focal/not radical | 183 (1.4%) | |
| Radical/not present Not radical | 48 (0.4%) | |
| Focal not radical Radical/not present | 261 (2%) | |
| Focal not radical Focal not radical | 21 (0.2%) | |
| Focal not radical Not radical | 5 (0%) | |
| Not radical N/A | 101 (0.8%) | |
| Missing | 5304 (41.4%) | |
| Social-economic status | | |
| Low | 3833 (29.9%) | |
| Middle | 4945 (38.6%) | |
| High | 3968 (31%) | |
| Missing | 67 (0.5%) | |
| Sentinel node procedure | | |
| Not performed | 3125 (24.4%) | |
| Performed | 7856 (61.3%) | |
| Missing | 1832 (14.3%) | |
| Result sentinel node procedure | | |
| Negative | 5141 (40.1%) | |
| ITC (≤ 0.2 mm) | 455 (3.6%) | |
| Micro metastases (> 0.2 mm, ≤2 mm) | 683 (5.3%) | |
| Positive (> 2 mm) | 1505 (11.7%) | |
| Not found | 215 (1.7%) | |
| Missing | 4814 (37.6%) | |
| Visits to GP | 16 ± 36 | |
| Complaints before diagnosis, 5 most common | 1. Uncomplicated hypertension (n=1233, 9.x6%)<br>2. Cystitis / other urinary infection (n=914, 7.1%)<br>3. Cough (n=794, 6.2%)<br>4. Upper respiratory infection acute (n=626, 4.9%)<br>5. Excessive ear wax (n=606, 4.7%) | |

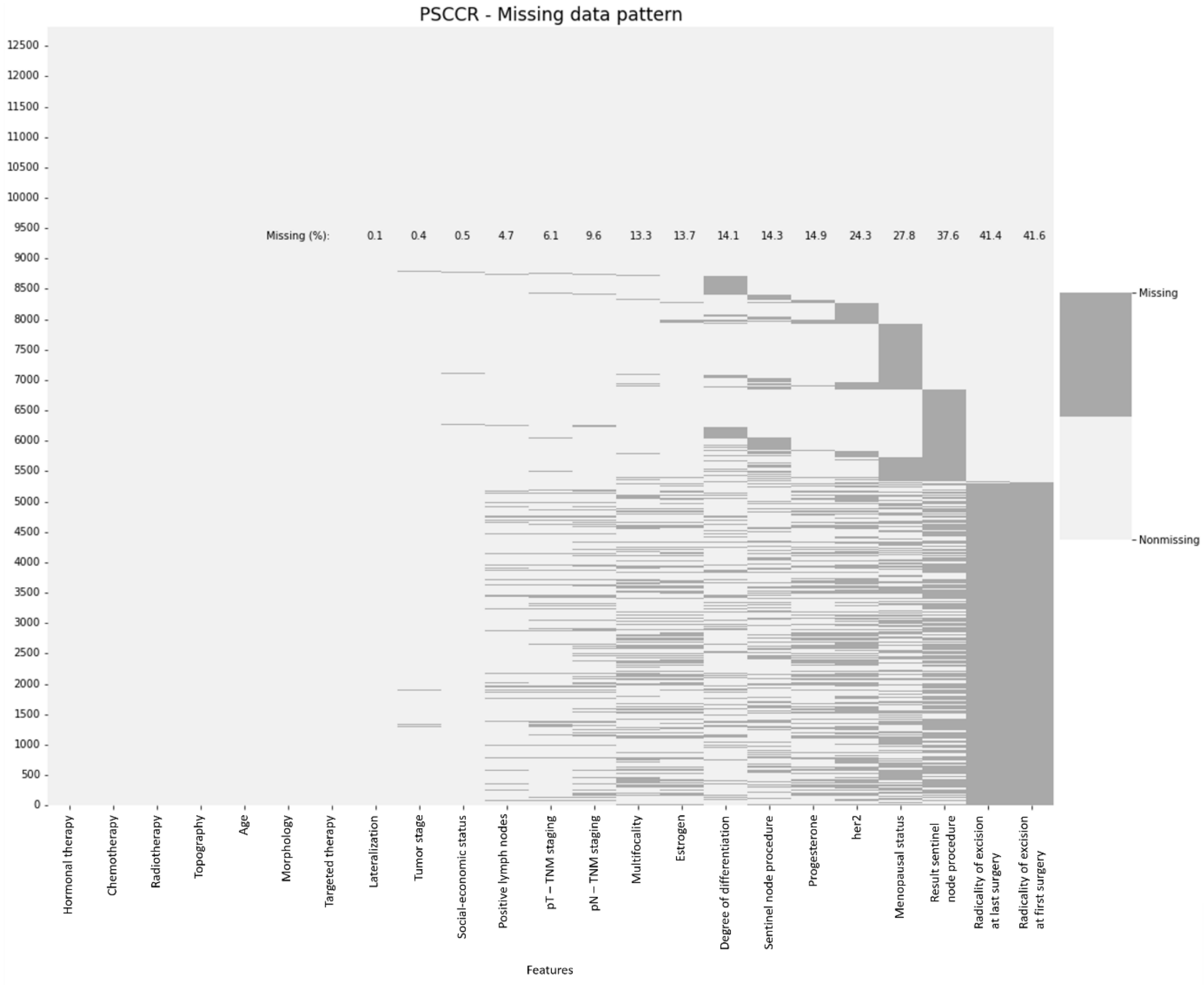


*Figure 4 – Missing data pattern for the PSCCR dataset. From left to right, more data is missing. The percentage of missing data is additionally displayed.*

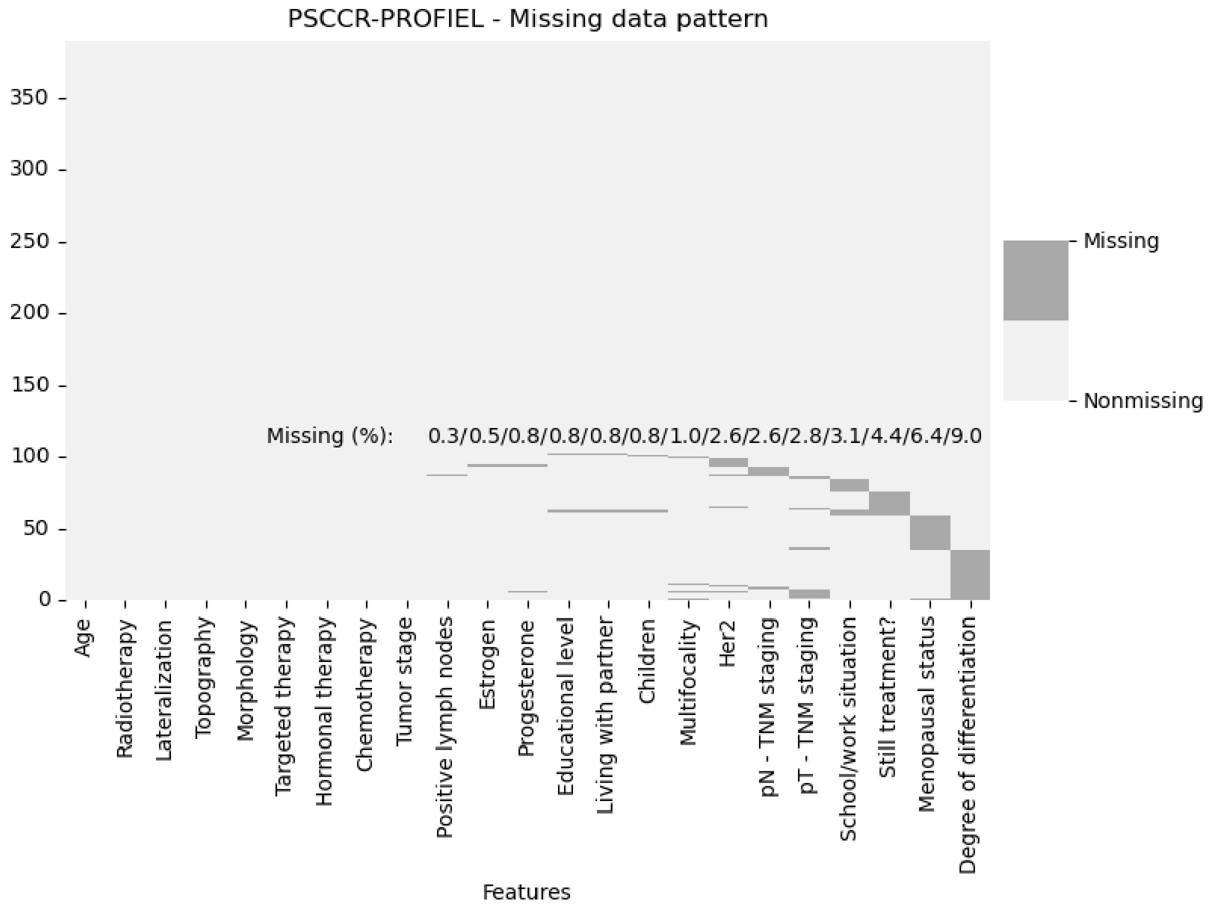


*Figure 5 – Missing data pattern for the PSCCR-PROFILES dataset. From left to right, more data is missing. The percentage of missing data is additionally displayed.*

*Table 2 – Model performance measured with Area Under the Curve (AUC) values for the various models and the various datasets. Instead of all predictors, the ten most important features (according to the RFC model) are selected. The values are the means and standard deviations over the five folds.*

| Ten variables | | AUC values | |
|---|---|---|---|
| | | PSCCR-PROFILES | PSCCR |
| Random Forest Classifier | Test<br>Train | 0.660 ± 0.032<br>0.807 ± 0.020 | 0.552 ± 0.015<br>0.836 ± 0.009 |
| Logistic Regression | Test<br>Train | 0.659 ± 0.040<br>0.688 ± 0.012 | 0.552 ± 0.006<br>0.564 ± 0.002 |
| Gaussian Naïve Bayes | Test<br>Train | 0.679 ± 0.035<br>0.693 ± 0.008 | 0.556 ± 0.010<br>0.566 ± 0.003 |
| K-Nearest Neighbors | Test<br>Train | 0.624 ± 0.079<br>0.867 ± 0.101 | 0.508 ± 0.018<br>0.797 ± 0.080 |
| Multi Layer Perceptron | Test<br>Train | 0.555 ± 0.103<br>0.563 ± 0.090 | 0.519 ± 0.017<br>0.527 ± 0.013 |

*Table 3 – Model performance measured with Area Under the Curve (AUC) values for the RFC and KNN models and the various datasets. Instead of all predictors, the six most important features (according to the RFC model) are selected. The values are the means and standard deviations over the five folds.*

| Six variables | | AUC values | |
|---|---|---|---|
| | | PSCCR-PROFILES | PSCCR |
| Random Forest Classifier | Test<br>Train | 0.674 ± 0.033<br>0.781 ± 0.008 | 0.533 ± 0.015<br>0.775 ± 0.006 |
| K-Nearest Neighbors | Test<br>Train | 0.620 ± 0.050<br>0.830 ± 0.079 | 0.511 ± 0.016<br>0.754 ± 0.065 |

*Table 4 – Model performance measured with Area Under the Curve (AUC) values for the RFC and KNN models and the various datasets. Instead of all predictors, the four most important features (according to the RFC model) are selected. The values are the means and standard deviations over the five folds.*

| Four variables | | AUC values | |
|---|---|---|---|
| | | PSCCR-PROFILES | PSCCR |
| Random Forest Classifier | Test<br>Train | 0.661 ± 0.038<br>0.763 ± 0.013 | 0.535 ± 0.010<br>0.701 ± 0.003 |
| K-Nearest Neighbors | Test<br>Train | 0.608 ± 0.040<br>0.787 ± 0.041 | 0.505 ± 0.015<br>0.670 ± 0.067 |

*Table 5 – Model performance measured with Area Under the Curve (AUC) values for the RFC and KNN models and the various datasets. Instead of all predictors, the two most important features (according to the RFC model) are selected. The values are the means and standard deviations over the five folds.*

| Two variables | | AUC values | |
|---|---|---|---|
| | | PSCCR-PROFILES | PSCCR |
| Random Forest Classifier | Test<br>Train | 0.664 ± 0.031<br>0.695 ± 0.009 | 0.518 ± 0.017<br>0.617 ± 0.008 |
| K-Nearest Neighbors | Test<br>Train | 0.617 ± 0.054<br>0.653 ± 0.035 | 0.503 ± 0.012<br>0.577 ± 0.009 |

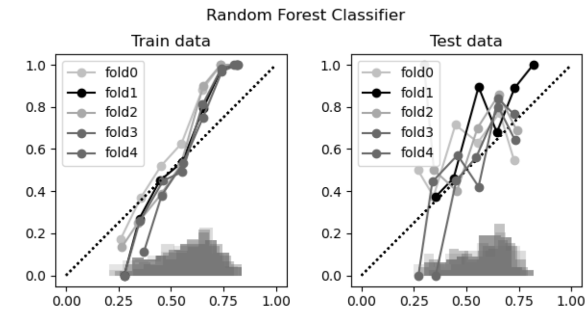


*Figure 6 – Calibration plot of all folds in the Random Forest Classifier on the PSCCR-PROFILES dataset*

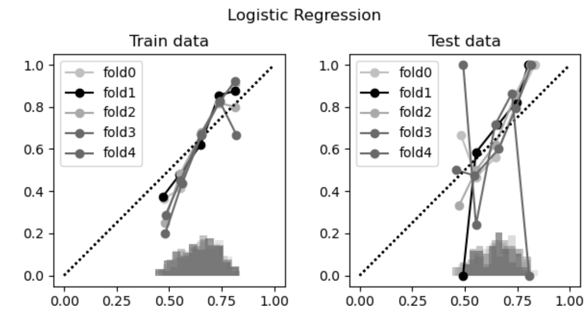


*Figure 7 – Calibration plot of all folds in the Logistic Regression model on the PSCCR-PROFILES dataset*

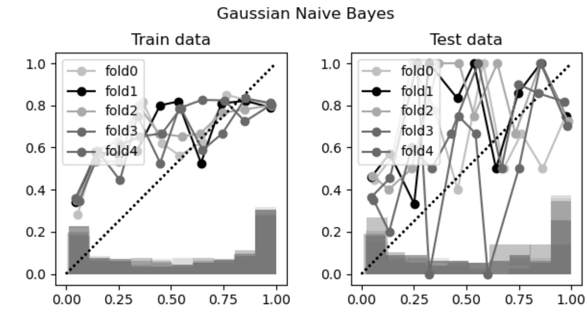


*Figure 8 – Calibration plot of all folds in the Gaussian Naïve Bayes model on the PSCCR-PROFILES dataset*

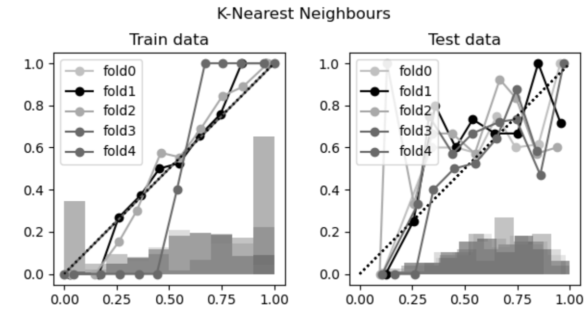


*Figure 9 – Calibration plot of all folds in the K-Nearest Neighbors model on the PSCCR-PROFILES dataset*

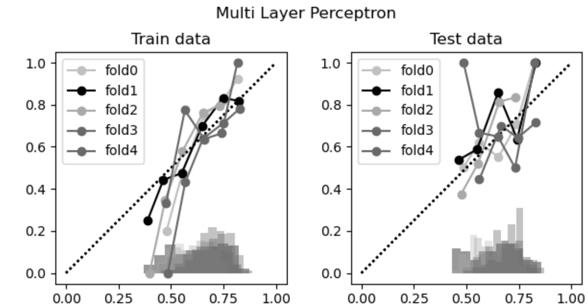


*Figure 10 – Calibration plot of all folds in the Multi Layer Perceptron on the PSCCR-PROFILES dataset*

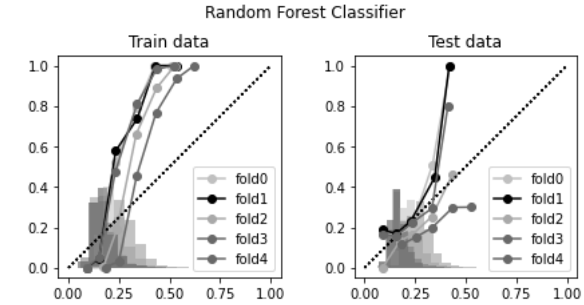


*Figure 11 – Calibration plot of all folds in the Random Forest Classifier on the PSCCR dataset*

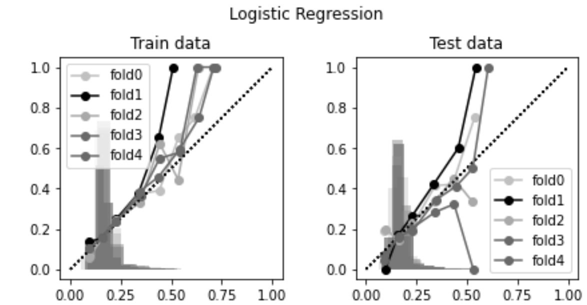


*Figure 12 – Calibration plot of all folds in the Logistic Regression model on the PSCCR dataset*

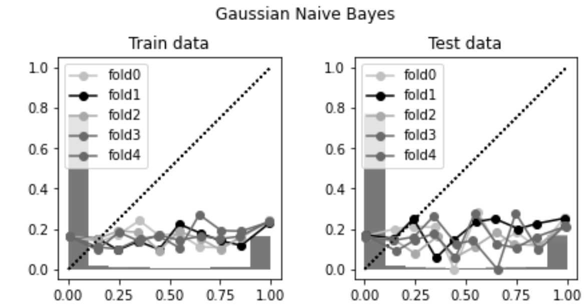


*Figure 13 – Calibration plot of all folds in the Gaussian Naïve Bayes model on the PSCCR dataset*

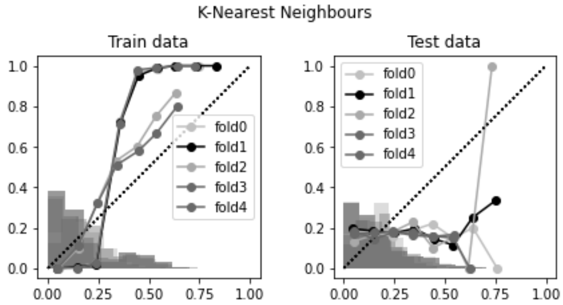


*Figure 14 – Calibration plot of all folds in the K-Nearest Neighbors model on the PSCCR dataset*

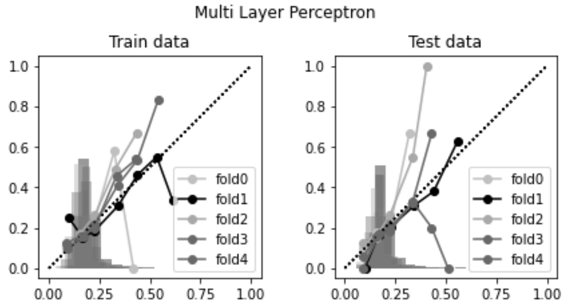


*Figure 15 – Calibration plot of all folds in the Multi Layer Perceptron on the PSCCR dataset*

## Online Resource 2: Python versions used

| | |
|---|---|
| Python | 3.9.7 |
| IPython | 7.29.0 |
| Spyder | 5.1.5 |
| Sklearn | 0.24.2 |
| Numpy | 1.20.3 |
| Pandas | 1.3.4 |
| Pickle | 4.0 |
| Statsmodels | 0.12.2 |
| Matplotlib | 3.4.3 |
| Miceforest* | 5.4.0 |
| Seaborn | 0.11.2 |
| Scipy | 1.7.1 |

* The package miceforest imputes data with a Random Forest. No further tuning to the ImputationKernel was done. The diagnostics plots within the miceforest package work for continuous variables and not for the categorical data we had in this study. Therefore, as an alternative, we visually compared the distribution over the categories before and after imputation. The choice for five imputed datasets was made because it was seen in literature most often, imputation was iterated for ten times.

## Online Resource 3: TRIPOD checklist

See pdf-file for page numbers where specific items are mentioned.

- Items 6b and 7b are not addressed in the manuscript. Both items are about blinding of assessment of predictors for the outcome and other predictors. For PSCCR and PSCCR-PROFILES, data was already collected in previous studies. Therefore, when Nivel data, NCR data and patient-reported outcomes were collected, neither of the people involved could possibly know of the link between the various predictors and the outcome fatigue. Therefore, no further actions were necessary to blind the assessment of the outcome (item 6b) and predictors for the outcome and other predictors (item 7b).
- Items 15a and 15b are not fully addressed in the manuscript. The items are about the model specification and as machine learning models were used, no further specifications as the regression coefficients and model intercept or baseline survival are known for these models. Additionally, as our results do not show at least acceptable discrimination, it is not possible to use the prediction model for individuals. Therefore, no further explanation is given on how to use the prediction model.
